# Alpine radish rhizosphere microbiome assembly and metabolic adaptation under PBAT/PLA humic acid biodegradable mulch films

**DOI:** 10.3389/fmicb.2025.1623052

**Published:** 2025-07-28

**Authors:** Jian Zhong, Ju Li, Jichao Liao, Yanqin Ma, Zhi Li, Liang Yang, Wei Chang, Mingjun Miao

**Affiliations:** ^1^Vegetable Germplasm Innovation and Variety Improvement Key Laboratory of Sichuan Province, Sichuan Academy of Agricultural Sciences, Horticulture Research Institute, Chengdu, China; ^2^Key Laboratory of Horticultural Crops Biology and Germplasm Enhancement in Southwest Regions, Ministry of Agriculture and Rural Affairs, Chengdu, China; ^3^Sichuan Institude of Edible Fungi, Chengdu, China

**Keywords:** alpine agriculture, biodegradable film, rhizosphere microbiome, metabolic adaptation, humic acid

## Abstract

**Introduction:**

Alpine agroecosystems present unique crop production challenges due to extreme environmental conditions, where rhizosphere microbiomes significantly influence plant adaptation.

**Methods:**

To investigate mulch-induced microbial changes in high-altitude agriculture, this study analyzed a radish field in China using SMRT sequencing (16S rRNA/ITS) and metagenomics, comparing PBAT/PLA biodegradable films with/without humic acid (HA) at varying thicknesses.

**Results:**

Results demonstrated that radish cultivation selectively enriched *Proteobacteria* and *Acidobacteriota* while depleting *Chloroflexi* and *Actinobacteria*, with fungal communities shifting from *Basidiomycota*-to *Ascomycota*-dominance. Notably, HA-amended mulches enhanced bacterial diversity and specifically promoted polymer-degrading microbes (*Chitinophagaceae, Candidatus_Udaeobacter, Chaetomiaceae*). Metagenomic profiling revealed thickness-dependent increases in functional genes related to carbohydrate and amino acid metabolism in HA-treated soils.

**Conclusion:**

These findings advance our understanding of how biodegradable mulch formulations can be optimized to enhance microbial ecosystem services in alpine farming systems.

## 1 Introduction

The rhizosphere microbiome is pivotal in plant health, nutrient cycling, and soil ecosystem stability, particularly in extreme environments such as alpine agroecosystems (Hou et al., 2024; Marian et al., 2022). Alpine soils are characterized by low temperatures, strong ultraviolet radiation, and nutrient limitations, and impose unique selective pressures on microbial communities (Ding et al., 2020). Radish (*Raphanus sativus* L.) is one of the most economical root vegetable crops widely cultivated in high-altitude regions and relies on its root-associated microbiome for stress resilience and nutrient acquisition (Kravchenko et al., 2022). Recent studies have highlighted the fact that rhizosphere microbial community structure and metabolic function can respond to certain environmental factors in crop growth, for example, *Claroideoglomus etunicatum* affects functional genes to aid maize cadmium and lanthanum stress (Hao et al., 2022; Jiao et al., 2023; Korenblum et al., 2022; Wu et al., 2024; Yue et al., 2023). However, the microbial ecology of high-altitude radish cultivation remains underexplored despite its economic importance and unique soil adaptation challenges.

Plastic mulch films [microplastics (MPs)] can retain soil moisture and heat retention and prevent weeds, and are widely used in agriculture to enhance crop production (Tian et al., 2022). China has the largest plastic mulch film usage worldwide, where plastic mulch film covers 19.8 million hectares of agricultural land (Liu et al., 2014; Qi et al., 2018). However, MP pollution has emerged as a critical environmental issue with extensive effects on soil ecosystems, particularly microbial communities (Fu et al., 2024; Yang et al., 2024). Concurrently, biodegradable mulches such as polybutylene adipate terephthalate/polylactic acid (PBAT/PLA) have been increasingly adopted to replace conventional plastics in sustainable agriculture (Xie et al., 2022). Humic acid (HA)-modified PBAT/PLA films are promising for enhancing microbial activity due to the dual role of HA as a carbon substrate and electron shuttle (Lu et al., 2023), yet their effects on rhizosphere microbial community structure and function have not been evaluated systematically, especially in alpine systems.

Recent advancements in high-throughput sequencing (HTS) have revolutionized microbial community analysis, enabling unprecedented resolution in microbiome studies. 16S rRNA and intergenic space (ITS) amplicon sequencing remain the gold standards for taxonomic profiling of bacteria and fungi, respectively, but the shift toward full-length reads (PacBio SMRT or Oxford Nanopore) has enhanced species-level classification and strain discrimination by overcoming short-read limitations (Davidson and Epperson, 2018; Sanschagrin and Yergeau, 2014; Shahi et al., 2019; Ye et al., 2020). Metagenomic sequencing transcends amplification biases, presenting functional insights via gene-centric analysis, pathway reconstruction, and metagenome-assembled genomes (Wu et al., 2022). Coupled with the application of bioinformatics, these technologies are pivotal for translational applications in human health, agriculture, and environmental monitoring.

In the present study, we combined 16S rRNA/ITS sequencing and Kyoto Encyclopedia of Genes and Genomes (KEGG) pathway analysis to characterize the alpine radish rhizosphere microbiome and evaluate how PBAT/PLA biodegradable mulch film modulates microbial community structure and shifts microbial metabolic activity.

## 2 Materials and methods

### 2.1 Experimental site and biodegradable film

The experimental site was a Plateau Radish Production Base in Ganzi Tibetan Autonomous Prefecture, Litang County, China (29°41′22″ N, 100°23′23″ E), which is 3637 m above sea level. This study used PBAT/PLA HA biodegradable silver black or black films and PBAT/PLA biodegradable black films as follows:

1. PBAT/PLA HA biodegradable silver black film (T1): 90 wt% PBAT, 5 wt% PLA, 5 wt% HA, 12-μm thickness;
2. PBAT/PLA HA biodegradable black film (T2): 90 wt% PBAT, 5 wt% PLA, 5 wt% HA, 8-μm thickness;
3. PBAT/PLA HA biodegradable black film (T3): 90 wt% PBAT, 5 wt% PLA, 5 wt% HA, 6-μm thickness;
4. PBAT/PLA biodegradable black film (T4): 90 wt% PBAT, 10 wt% PLA, 8-μm thickness;
5. PBAT/PLA biodegradable black film (T5): 90 wt% PBAT, 10 wt% PLA, 6-μm thickness.

All films were purchased from Qingtian Plastic Co., Ltd.

### 2.2 Experimental design

Radishes (radish seed variety: Xiashuai 335; company: Zhengzhou Harvest Seed Co., Ltd.) were sown directly into the soil on July 20, 2023, and covered with the five mulch films (T1–5). The blank control was uncovered soil (CKw). Each treatment was performed three times. Soil samples were collected from the top 0–20 cm of the experimental plots using the five-point sampling method when the radishes were harvested on September 20, 2023. The soil pre-radish sowing was sampled in the same manner (CKq). A total of 105 soil samples (each 100 g) were collected and divided into seven groups according to the sample source after sieving and mixing, and stored at 80°C for microbial community structure and function analyses.

### 2.3 Analysis of soil microbial communities

The soil samples were extracted to analyze the bacterial and fungal communities based on single-molecule real-time (SMRT™) DNA sequencing technology. Total DNA was extracted from the seven soil pools using the cetyltrimethylammonium bromide–sodium dodecyl sulfate (CTAB–SDS) method. The bacterial and fungal communities were analyzed by amplifying the 16S rRNA and ITS genes, respectively, of distinct regions with specific primers tagged with barcodes. The PCR was conducted using TransStart^®^ FastPfu DNA Polymerase (TransGen Biotech). Sequencing libraries were generated using a SMRTbell™ Template Prep Kit (PacBio) following the manufacturer's recommendations. The library quality was assessed on a Qubit 2.0 fluorometer (Thermo Fisher Scientific) and Femto Pulse system. Finally, the library was sequenced on the PacBio Sequel platform. All library preparation and sequencing procedures were performed by Novogene (China).

### 2.4 Analysis of soil microbial community function

Total DNA was extracted using the CTAB–SDS method, and the genomic DNA sample was fragmented into short fragments for metagenome sequencing. These DNA fragments were end-polished, A-tailed, and ligated with full-length adapters for Illumina sequencing before size selection. Subsequently, PCR amplification was performed, and purification was conducted through an AMPure XP system (Beverly). The resulting library was assessed on an Agilent Fragment Analyzer System (Agilent) and quantified to 1.5 nM through Qubit (Thermo Fisher Scientific) and quantitative PCR. The qualified libraries were pooled and sequenced on Illumina platforms according to the effective library concentration and data amount required. Novogene performed all library preparation and sequencing procedures.

### 2.5 Data analyses

The 16S rRNA/ITS sequencing reads were trimmed in the PacBio SMART portal to remove low-quality reads, then truncated by cutting off the barcode and primer sequence for further analysis. Clean reads were obtained by removing chimera sequences through comparison with the reference database using the UCHIME algorithm (http://www.drive5.com/usearch/manual/uchime_algo.html). Then, the representative sequence for each operational taxonomic unit (OTU; clean reads with 97% similarity were assigned to the same OTUs) was produced using UPARSE (v7.0.1001) and assigned to the small subunit (SSU) rRNA databases of the SILVA and UNITE databases to complete the species annotation. Species abundance was normalized using a standard of sequence number corresponding to the sample with the most sequences.

Clean data were obtained by pre-processing the Illumina sequencing reads using Fastp (https://github.com/OpenGene/fastp), then *de novo* assembled using MEGAHIT with the following parameters: -presets meta-large (-end-to-end, -sensitive, -I 200, -X 400). Redundancy was eliminated using CD-HIT software and the unique gene was obtained. Subsequently, unigenes were aligned with those in the KEGG database using DIAMOND software (https://github.com/bbuchfink/diamond/) with the following parameter settings: blastp, -e 1e-5. The best blast hit results from the alignment results of each gene were selected for subsequent analysis. The abundance of each gene was estimated as reads per kilobase per million mapped reads (RPKM) using BBMap. Data with RPKM ≤ 10 were removed to prevent false positives.

The Shannon–Wiene index is an important indicator of alpha-diversity and is used to demonstrate the observed richness or evenness of microbial species within-group. Similarities or differences among the samples were visualized using principal co-ordinate analysis (PCoA) with the Bray-Curtis algorithm. The species or functional gene differences between groups were searched using metagenomeSeq and linear discriminant analysis (LDA) effect size (LEfSe) analyses. metagenomeSeq analysis involves permutation testing between groups on each taxonomy level and obtains a *p*-value. The LEfSe was conducted using LEfSe software (default LDA score: 4). The other related indices in the samples were calculated with QIIME (version 1.9.1) and displayed with R (version 2.15.3).

## 3 Results

### 3.1 Overview of alpine radish rooting soil microbial community

#### 3.1.1 Bacterial community structure

**Figure 1:**
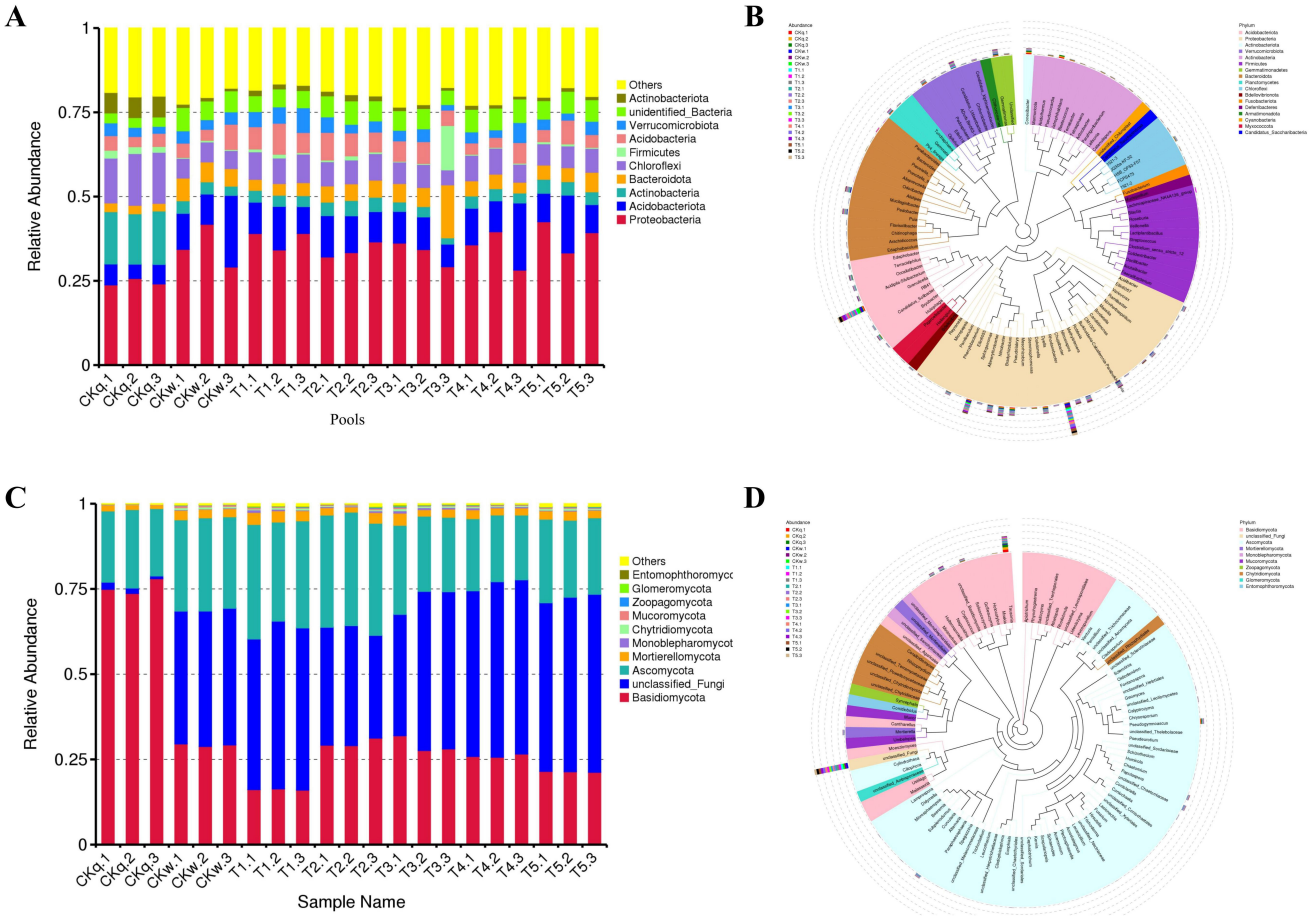
Overview of the radish rhizosphere microbiome. **(A)** The top 10 bacterial compositions of each pool at phylum level. **(B)** Evolutionary tree of the top 100 bacterial genera abundances. **(C)** The top 10 fungal compositions of each pool at phylum level. **(D)** Evolutioary tree of the top 100 bacterial genera abundances.

The 16S rRNA sequencing generated 5244 OTUs, which were assigned to 66 known phyla and 332 families. *Proteobacteria, Acidobacteriota, Chloroflexi, Actinobacteria, Bacteroidota*, and *Verrucomicrobiota* were the most abundant bacterial phyla in the alpine crop soil. Compared to the pre-planting soil, the radish rooting soil had significantly increased abundances of *Proteobacteria, Acidobacteriota, Bacteroidota, Actinobacteria*, and *unidentified_Bacteria*, while the abundances of *Chloroflexi, Actinobacteria*, and *Firmicutes* were reduced (Figure 1A). Furthermore, 556 known genera were identified, where *Granulicella, Rhodanobacter, Sphingomonas, Conexibacter, Candidatus_Udaeobacter, Pseudonocardia*, and *Burkholderia-Caballerronia-Paraburkholderia* were the dominant genera in the alpine radish rooting soil (Figure 1B).

#### 3.1.2 Fungal community structure

The ITS sequencing produced 1,899 OTUs, which were divided into 19 known phyla and 226 families. *Basidiomycota, Unclassified_Fungi, Ascomycota*, and *Mortierellomycota* were the predominate phyla in the alpine crop soil. The abundance of *Basidiomycota* decreased markedly after the radish planting, while the abundance of *Unclassified_Fungi* and *Ascomycota* increased (Figure 1C). The analysis assigned 357 known genera, among which *Tausonia, Unclassified_Fungi, Alternaria, Pseudogymnoascus, Cryptococcus, Unclassified_Chaetomiaceae, Solicoccozyma, Naganishia*, and *Papulaspora* were the dominant genera in alpine crop soil (Figure 1D).

### 3.2 Microbial diversity of alpine radish rooting soil under PBAT/PLA biodegradable mulch film

#### 3.2.1 Alpha-diversity

**Figure 2:**
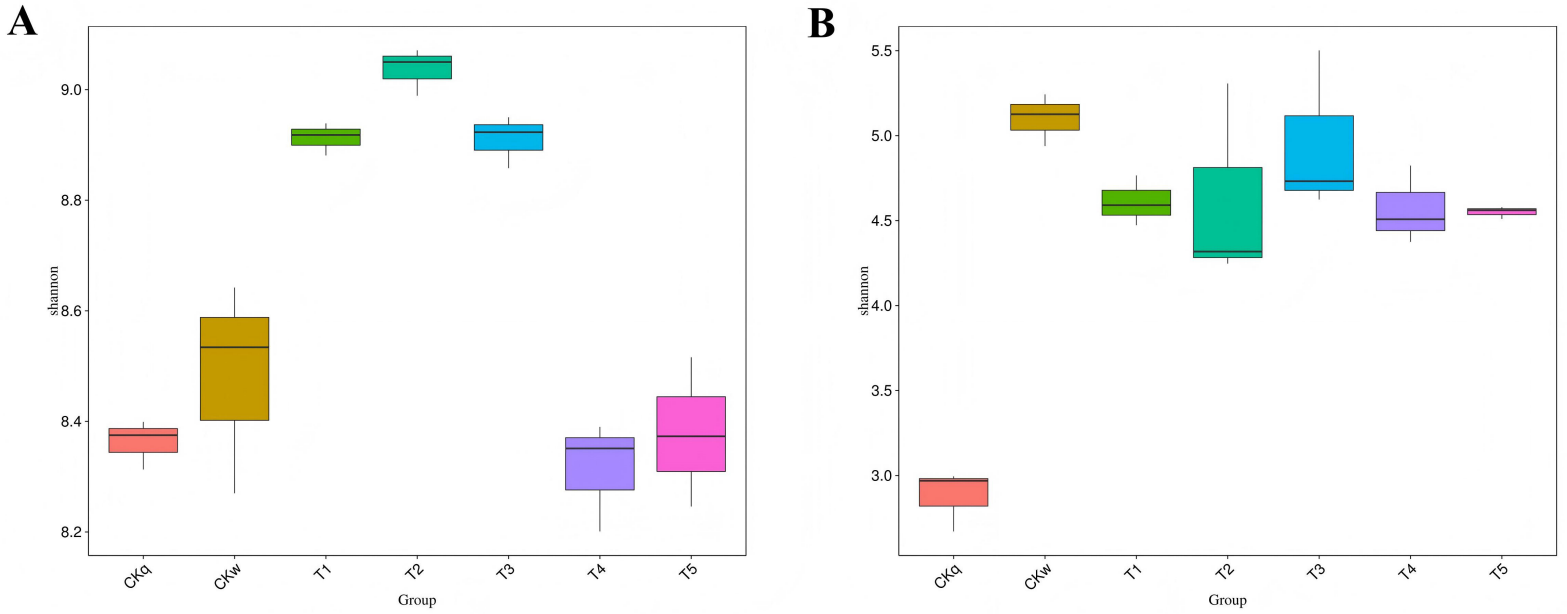
Comparison of alpha-diversity (Shannon–Wiener index) based on OTUs. **(A)** Bacterial community diversity. **(B)** Fungal community diversity.

The microbial diversity of the mulch treatment groups was compared based on the OTU number and abundance. The bacterial community alpha-diversity index was highest in the PBAT/PLA HA biodegradable film groups (Shannon-Wiener index >8.8), whereas the PBAT/PLA biodegradable film groups had a similar bacterial community alpha-diversity index to that of the no-mulch group. The fungal community alpha-diversity index did not differ significantly among the mulch treatment groups (Figure 2).

**Figure 3:**
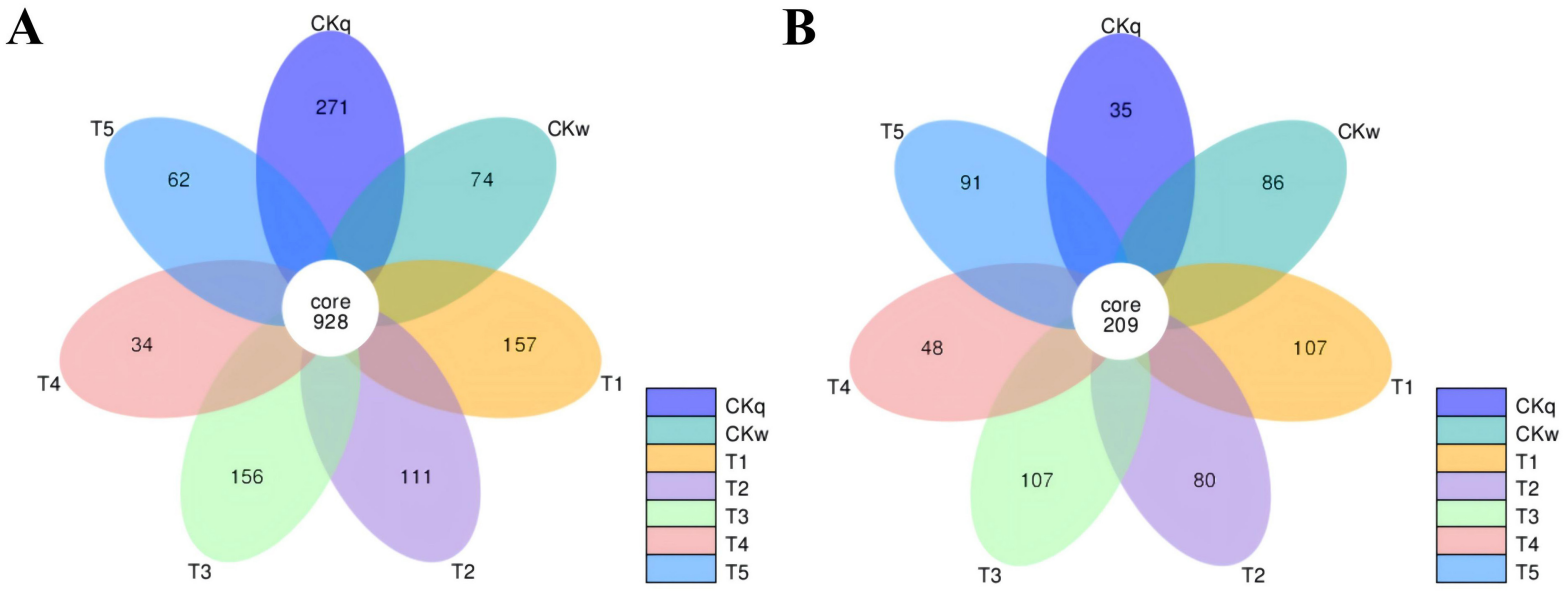
Venn diagrams of the microorganisms shared between mulch film-treated groups. **(A)** Bacteria. **(B)** Fungi.

There was a majority of overlapping microbial OTUs between the mulch treatment groups. Overall, the number of microbial OTUs specific to PBAT/PLA HA biodegradable film was approximately twice as high as that in the other mulch treatment groups. Notably, the PBAT/PLA HA biodegradable film had a greater effect on the fungal community structure in the radish rooting soil, where the number of unique fungal OTUs accounted for almost half of the total number of fungal OTUs in the mulch treatment groups (Figure 3).

#### 3.2.2 Beta-diversity

**Figure 4:**
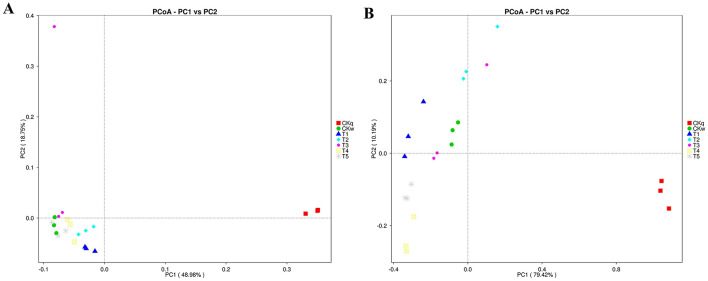
PCoA (applying Bray-Curtis algorithm) for microbe composition according to mulch film. **(A)** Bacteria. **(B)** Fungi.

**Figure 5:**
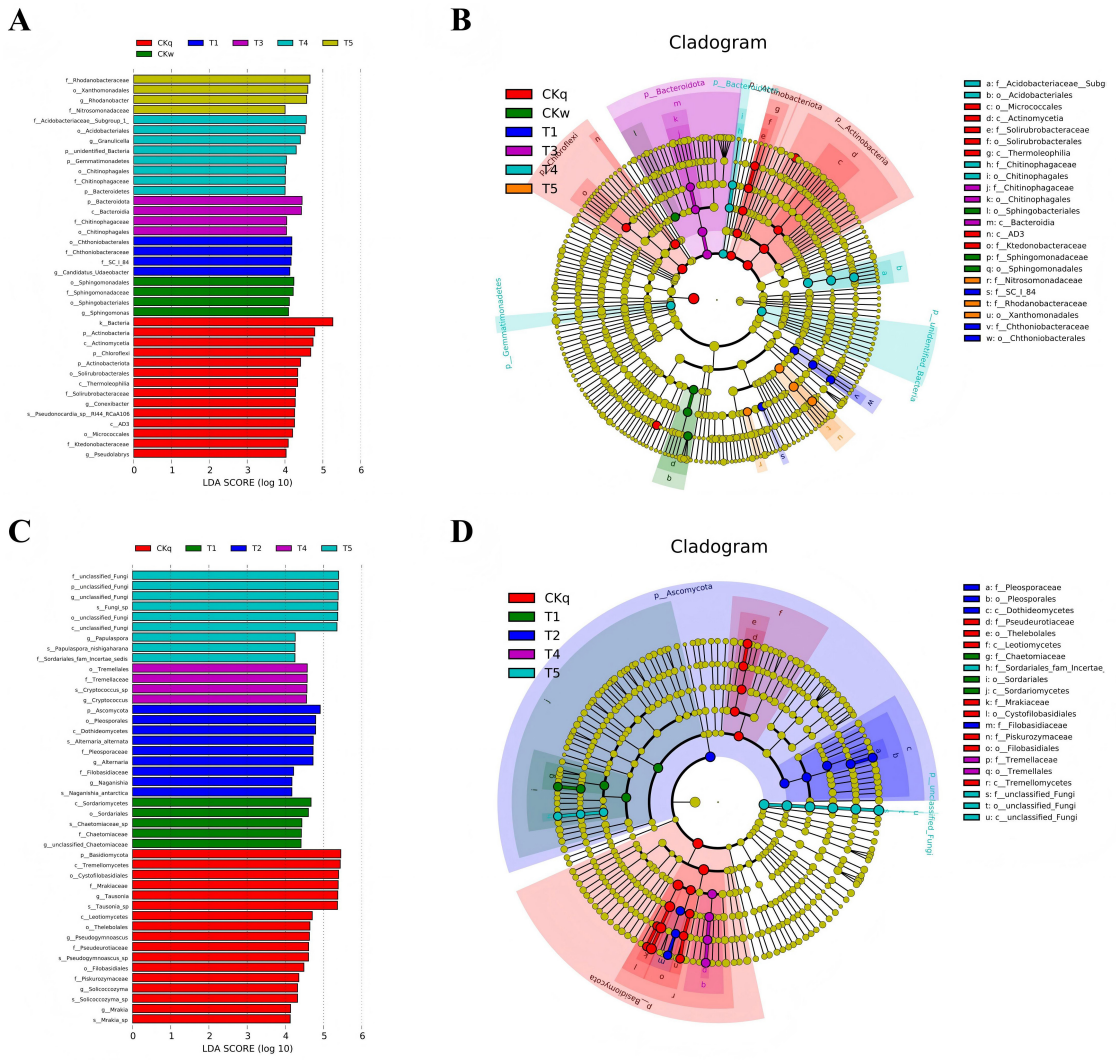
Marker species with significantly different abundances in the mulch treatment groups. **(A)** LEfSe results for bacteria. The histogram depicts species with LDA score differences >4. **(B)** Evolutionary tree of bacteria based on marker species identified. **(C)** LEfSe results for fungi. **(D)** Evolutionary tree of fungi based on marker species identified.

The effect of the mulch films on microbial composition was assessed using PCoA. The results indicated that the PBAT/PLA HA biodegradable films affected the bacterial communities, while more pronounced effects were observed in the fungal communities in all mulch treatment groups (Figure 4). Microbial species with significant differences among the mulch treatment groups were determined using LEfSe analysis, which detected 38 and 44 significantly differential microbial species in the bacterial and fungal community, respectively. In the PBAT/PLA HA biodegradable film group, the marker bacteria were *Chitinophagaceae, Chthoniobacteraceae*, and *Candidatus_Udaeobacter* (a group of bacteria involved in the natural carbon and nitrogen cycle and in degrading soil organic matter) and *SC_84*, while the marker fungal groups in the radish rooting soil fungal community were *Chaetomiaceae* and *Filobasidiaceae* (Figure 5).

### 3.3 Microbiome activity in radish rooting soil under PBAT/PLA biodegradable mulch films

**Figure 6:**
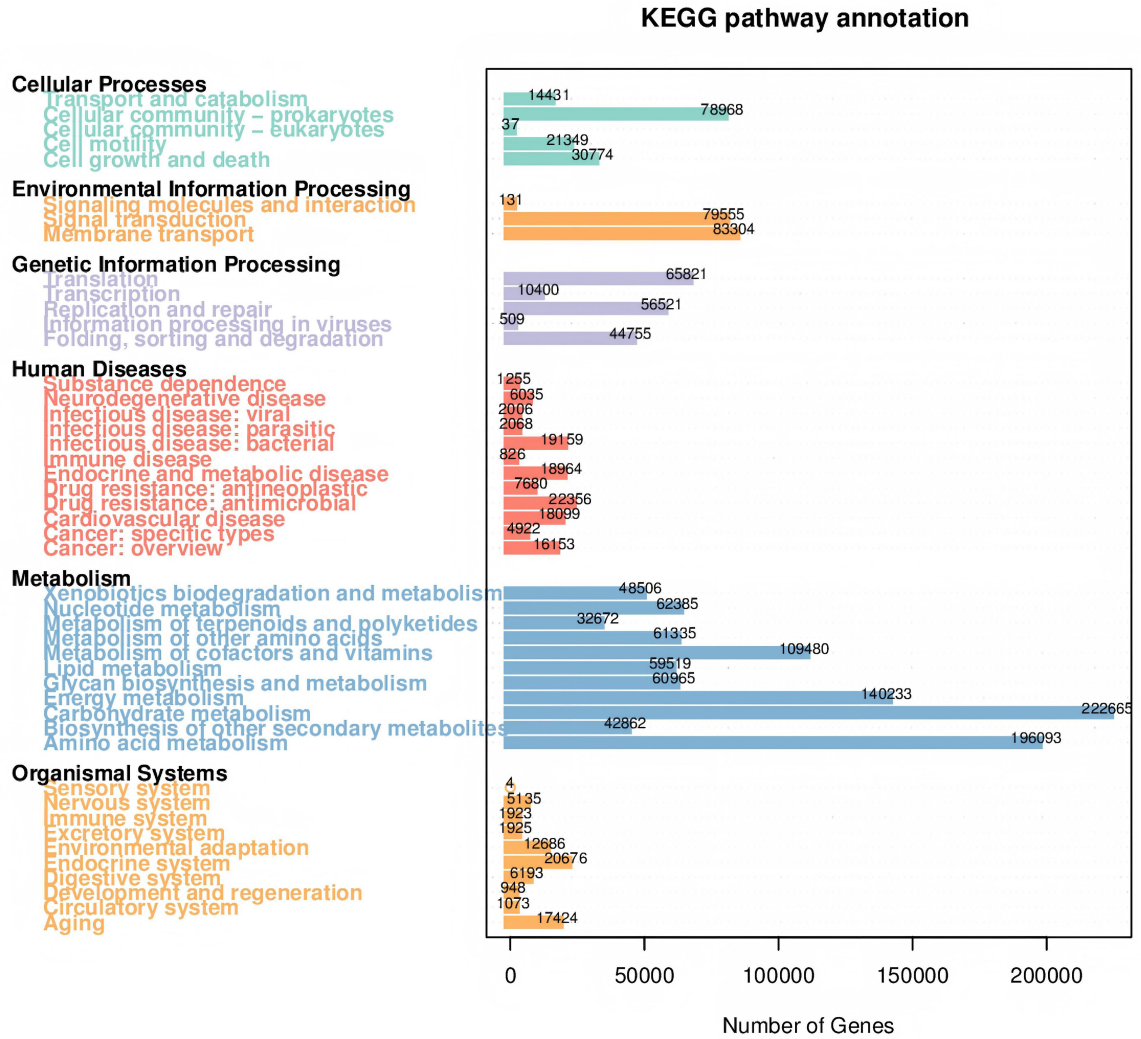
Total KEGG pathways identified in all soil pools.

**Figure 7:**
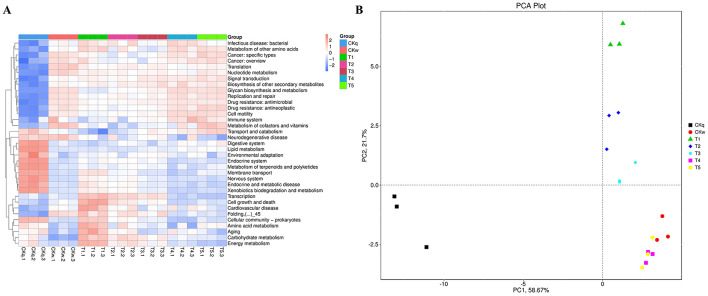
Metabolic differences in mulch treatment groups. **(A)** Heatmap depicts the normalized abundance of each KEGG pathway under the mulch treatments. **(B)** PCA of metabolic pathway composition according to mulch film.

**Figure 8:**
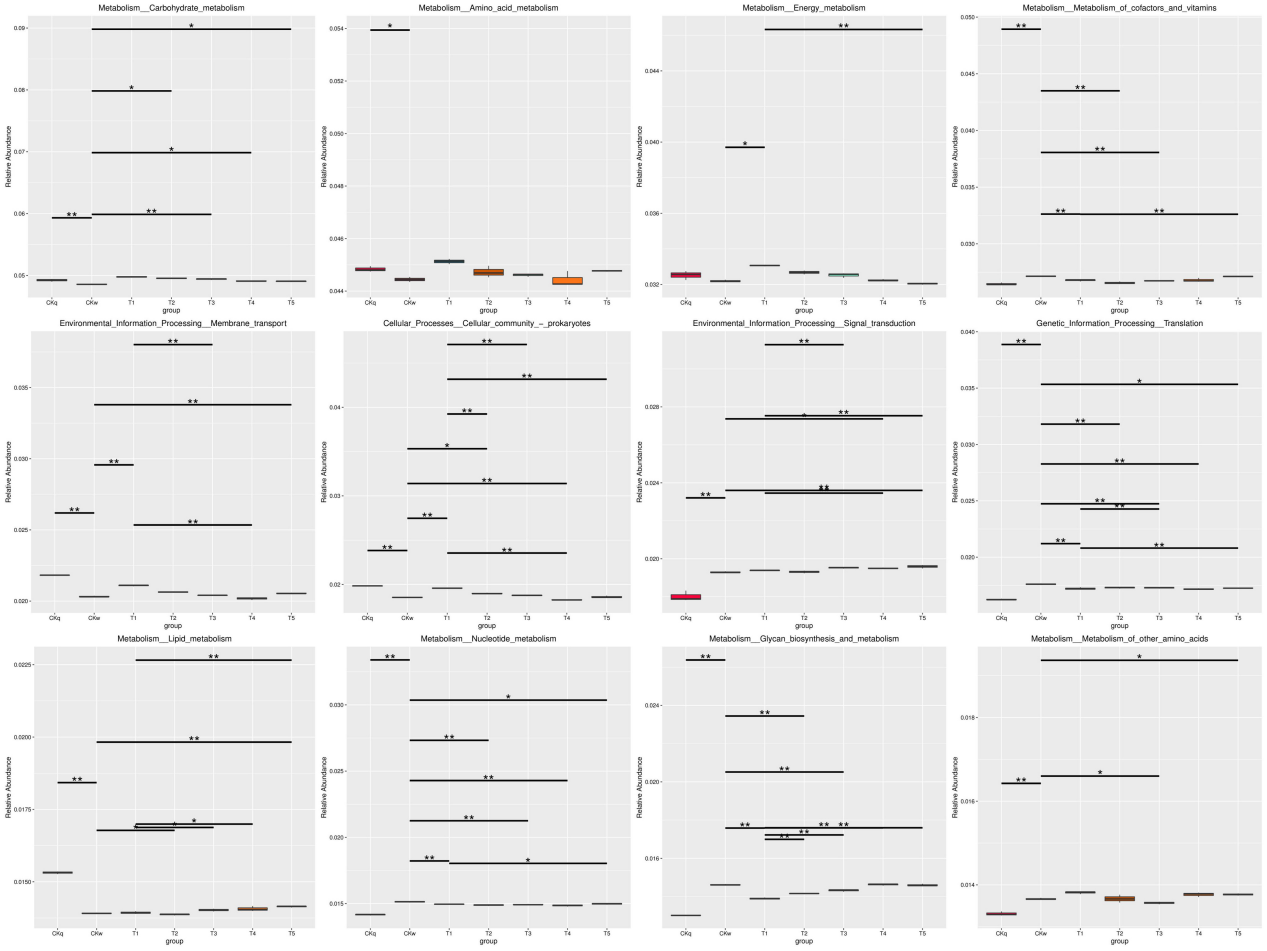
MetagenomeSeq analysis results for marker functional genes.

An average 375,137 contiguous sequences (contigs) were assembled in each sample and annotated to six sections and 46 domains of the KEGG pathways. Metabolism was the predominant microbiota functional activity, where carbohydrate fumetabolism, amino acid metabolism, energy metabolism, and metabolism of co-factors and vitamins accounted for 65% of all metabolic pathways (Figure 6). The results revealed a similar microbial activity composition in the PBAT/PLA biodegradable film and no-film groups, while the PBAT/PLA HA biodegradable film groups had similar microbial activity compositions, with higher functional activities such as metabolism (amino acid, carbohydrate, energy), cellular processes, and genetic information processing (cellular community-prokaryotes, cell growth and death, transcription, folding, sorting and degradation) (Figure 7). The metagenomeSeq analysis identified 12 differing functional genes, and the PBAT/PLA HA biodegradable film groups had higher abundances of carbohydrate metabolism, amino acid metabolism, energy metabolism, and cellular community_prokaryotes. Furthermore, decreasing the mulch film thickness tended to decrease the abovementioned abundances slightly (Figure 8).

## 4 Discussion

### 4.1 Features of radish rooting microbial community structure and ecological significance

In the present study, HTS technology was used to reveal for the first time the structure of the alpine radish root microbial community and microbial metabolic response to PBAT/PLA biodegradable mulch, providing important microbiological insights for sustainable high-altitude agricultural development. The results revealed that the alpine radish rhizosphere exhibited a distinct microbial community structure dominated by *Proteobacteria, Acidobacteriota, Chloroflexi, Actinobacteria, Bacteroidota*, and *Verrucomicrobiota*, as discovered by Huang and Eilers, *Proteobacteria* and *Actinobacteria* are specific microbes for cruciferous vegetable and cold-hardy plants, respectively (Huang et al., 2019; Eilers et al., 2010), suggesting strong plant–microbe interactions in high-altitude agroecosystems. The enrichment of *Proteobacteria* and *Acidobacteriota* in the rhizosphere aligned with their roles in carbon cycling and root exudate metabolism observed by Dey in low-altitude crops (Dey, 2017). However, the significant decline in *Chloroflexi* and *Actinobacteria* supports the “niche exclusion due to competitive root colonization” hypothesis proposed by Jacoby, though the high-altitude environment may intensify this competitive effect (Jacoby and Kopriva, 2019). Additionally, the prevalence of *Granulicella* and *Rhodanobacter* (which exhibit acid tolerance and sulfur metabolism capabilities) suggests a unique adaptation to alpine soil conditions, a phenomenon not previously reported in low-altitude radish cultivation systems. The fungal communities were structured by *Basidiomycota, Unclassified_Fungi, Ascomycota*, and *Mortierellomycota* and significantly shifted from *Basidiomycota* (saprotroph*s*) to *Ascomycota* (diverse symbionts/pathogens), suggesting that radish roots may selectively recruit beneficial fungi while suppressing wood-degrading taxa. The high proportion of *Unclassified_Fungi* highlighted the unexplored fungal diversity in alpine soils, which warrants metagenomic binning efforts. Studies have demonstrated that plant species can modulate active microbiota diversity through root exudation and environmental adaptive responses (Achouak et al., 2019; Perez-Mon et al., 2022). Therefore, further investigation into the combined effects of radish metabolome and alpine environmental factors on radish rhizosphere microbiota is warranted.

### 4.2 Effects of PBAT/PLA biodegradable films on soil microbial diversity and composition

Given their unique characteristics of particle size, polymer type, additive composition, and content, mulch films have positive, negative, or no significant effects on soil microbial community abundance and diversity (Feng et al., 2022; Ke Li, 2025; Xu et al., 2024). However, MP pollution typically disrupts the microbial community composition in the soil surrounding plant roots. Nevertheless, HA counteracts the harmful effects of MPs on the rhizosphere soil microbial community composition (Virachabadoss et al., 2024). Similarly, our results revealed that the PBAT/PLA HA biodegradable films enhanced bacterial diversity and fostered distinct microbial consortia, likely due to the dual role of HA as a carbon source and microbial activity stimulant. Furthermore, we identified the marker bacteria *Chitinophagaceae* and *Candidatus_Udaeobacter*, which degrade complex polymers and may have accelerated mulch degradation by secreting hydrolytic enzymes (chitinase, esterase) and contributed to soil carbon cycling (Bandopadhyay et al., 2020; Eichorst et al., 2013; Hansen et al., 2023) following the application of the PBAT/PLA HA biodegradable films. In contrast, fungal communities were more sensitive to all mulch film types, possibly due to hyphal network disruption by film residues. *Chaetomiaceae* are a key fungal taxon for lignocellulose degradation, and their laccase gene is highly expressed in composted soils. Furthermore, *Chaetomium globosum* can degrade polyurethane by secreting esterases and peroxidases, which is a similar mechanism applicable to other polyester-based plastics such as PBAT (Liu et al., 2024; Qian et al., 2024). Therefore, the soil microbial composition was responsive to the physical and chemical properties of the PBAT/PLA HA biodegradable films, which enhanced soil microbial–plant interactions through film degradation.

### 4.3 Effects of PBAT/PLA biodegradable films on soil microbial functions

The functional profiling of microbial communities in the radish rooting soil revealed significant metabolic adaptations in response to the PBAT/PLA biodegradable mulch films. The dominance of metabolic pathways (65% of total KEGG annotations), particularly carbohydrate, amino acid, and energy metabolism, aligned with previous studies demonstrating that polymer-degrading microbiomes prioritize core metabolic functions to utilize mulch-derived substrates (Zhong and Agarwal, 2024). Notably, the PBAT/PLA HA films enhanced functional activity compared to conventional PBAT/PLA films and bare soil, suggesting that HA may stimulate microbial metabolism through two mechanisms: (1) as a supplementary carbon source that co-metabolizes with mulch components, or (2) it improves soil physicochemical properties to facilitate microbial access to polymeric substrates (Li et al., 2022). The distinct functional signature of the HA-modified films was characterized by elevated carbohydrate metabolism and cellular community functions, indicating a more robust microbial network capable of coordinated mulch degradation. This result was supported by the metagenomeSeq data that demonstrated enriched genes for extracellular hydrolases and prokaryotic cell–cell interaction pathways.

The observed thickness-dependent decline in metabolic gene abundance may reflect substrate limitation in thinner films, consistent with a previously proposed carbon-use efficiency theory (Sinsabaugh et al., 2017). These results advance our understanding of how mulch formulation affects soil microbiome functionality. However, the thickness correlation introduced new considerations for agricultural practice: while thicker films may sustain metabolic activity, optimal thickness must balance degradation rates with crop cycle durations. Future studies should quantify the temporal dynamics of these metabolic shifts and their association with mulch mineralization rates.

## 5 Conclusions

This study highlights the critical role of rhizosphere microbiome modulation in enhancing crop resilience in high-altitude agroecosystems. The incorporation of humic acid into PBAT/PLA biodegradable mulch films emerges as a promising strategy for sustainable alpine agriculture, demonstrating dual benefits in both enriching functional microbial taxa and stimulating metabolic pathways essential for plant adaptation. Our findings provide a scientific foundation for developing next-generation biodegradable mulches that are specifically tailored to optimize soil microbiome functions under extreme environmental conditions.

## Data Availability

The original contributions presented in the study are publicly available. This data can be found in here: https://www.ncbi.nlm.nih.gov/, accession numbers PRJNA1295589 (Metagenomic data), PRJNA1296783 (ITS data) and PRJNA1296803 (16S rRNA data).
